# Confronting Consumers’ Complicity: Do Confrontations with Causal Responsibility for Sweatshop Labor Raise Moral Obligation?

**DOI:** 10.5334/irsp.775

**Published:** 2024-02-27

**Authors:** Felicitas Flade, Mario Messer, Roland Imhoff

**Affiliations:** 1 Johannes Gutenberg University, Mainz, Germany; 2 University of Cologne, Germany

**Keywords:** moral judgment, moral obligation, confrontation, causal responsibility, sweatshops

## Abstract

We report an internal reanalysis of five exploratory studies (total *N* = 1460) and two preregistered experiments (*Ns* = 778; 528), in which we investigated to what extent perceived causal involvement in harming sweatshop workers increases perceived moral obligation to support the workers. Within hypothetical scenarios as well as alleged magazine articles, target persons purchasing sweatshop-made products were contrasted with uninvolved bystanders. When participants made judgments about abstract others, causal involvement moderately increased ratings of moral obligation. However, when facing their own complicity in maintaining sweatshop conditions, the effect of causal involvement was small to non-existent. The greater sensitivity to the moral imperative of causal responsibility for indirect harm within global supply chains for others than for the self cannot be attributed to defensive processes, however. To the contrary, moral obligation for the self remained comparatively high, even if causal responsibility was low, presumably due to the greater reliance on internal states for the self.

Few realize that severe poverty is an ongoing harm we inflict upon the global poor. If more of us understood the true magnitude of the problem of poverty and our causal involvement in it, we might do what is necessary to eradicate it.

Pogge, 2005 (p. 1)

Imagine walking through a shopping street and being approached by an anti-poverty activist who explains that as a consumer of fashion you are contributing to the perpetuation of poor working conditions and hence involved in causing the suffering of garment workers in the global South. How much do you feel morally obligated to support the suffering workers? Now, imagine merely witnessing how the activist delivers the same argument to another person. How much do you think this person should feel morally obligated to act in this case? In the present paper, we sought to tackle this issue empirically in two scenario-based experiments. Specifically, we tested to what extent confrontation with one’s causal involvement in an exploitative global supply chain increases the moral obligation to act against poverty and human rights violations. We also investigated whether causal involvement has the same effect when people decide about *others*’ moral obligation, and consider the role of salience of internal states on the relationship between causal involvement and moral obligation judgments.

## Moral Obligation Is a Key Antecedent of Increased Action Against Global Injustice

To understand why the *advantaged* act in favor of disadvantaged groups, it is essential to consider moral motivations (van Zomeren et al., 2011). Moral motivations predict collective action (Sabucedo et al., 2018), helping the poor (González & Lay, 2017), volunteering (Ellemers & Boezeman, 2010), and the purchase of fair-trade products (O’Connor et al., 2017). When moral beliefs (e.g., values or moral convictions) evoke action against injustice, this effect is mediated by a heightened sense of *moral obligation* as a proximal antecedent of behavior (Sabucedo et al., 2018). Moral obligation, the felt motivation to act in concordance with one’s moral self-expectations (Schwartz & Howard, 1981), predicts behavioral intentions in the case of actions that have consequences for the welfare of others (Rivis et al., 2009), like choosing a product that promotes good working conditions (O’Connor et al., 2017). When it comes to political action, moral obligation increases the intention to protest as well as actual participation in a demonstration (Sabucedo et al., 2018).

However, raising feelings of moral obligation to act against sweatshop conditions in global supply chains proves to be a challenge. Because it occurs within a complex global system of political and economic interdependencies, the suffering of factory workers in the global South lacks important characteristics that are crucial for the activation of moral obligation (Lichtenberg, 2010). For example, there is no personal contact involved, salience of need is low, victims are abstract, victims are members of an outgroup, perceived efficacy is low, and many other potential helpers are present (Lichtenberg, 2010; Schwartz, 1977). In short, the situation lacks psychological proximity (Ghorbani et al., 2013).

## Facing One’s Own Involvement in International Exploitation

Feelings of moral obligation among those in the global North may be increased by highlighting their causal involvement in creating and maintaining global poverty (Lichtenberg, 2010; Pogge, 2005, 2011) and the negative consequences of their lifestyle for people in the global South (Rosenmann et al., 2016). Customer demand feeds global supply chains connecting consumers in the wealthier countries with those who produce the goods for global retailers in developing countries. In the case of the global fashion industry, it may be argued that consumers are not only bystanders witnessing the suffering of distant others, but that they are causally involved in harming those on the other end of the supply chain by their consumption choices (Barnes & Lea-Greenwood, 2006; Merk, 2014; Taplin, 2014).

Indeed, how people explain the existence of poverty in the global South is critical for their motivation to help (González & Lay, 2017). When they perceive it to be caused by international exploitation, they are more willing to act (Pinazo et al., 2010). Focusing on third-party perpetrators such as governments raises moral outrage and motivates political action (Thomas & McGarty, 2018). Generally, when observers perceive *others* as having caused a foreseeable harm, they assign blame and call for compensation (Darley & Pittman, 2003). By contrast, uninvolved bystanders are seen as less morally obligated to act, because people judge failures to help (omissions) as less immoral than harmful actions (Kordes-de Vaal, 1996). Clearly, causal responsibility for harm matters when it comes to the moral obligation to help.

Again, moral judgments about the *causal involvement of individual actors* (e.g., consumers) in doing harm within complex systems such as global supply chains are understood less well. In the case of buying sweatshop-made products, harm is carried out indirectly involving countless other contributors (Lichtenberg, 2010), thus evoking more lenient moral judgments (Paharia et al., 2009). Hence, the effect of involvement in doing harm on judgments of moral obligation to help may be reduced compared to more direct situations.

## Can Perceived Causal Responsibility Activate the Moral Obligation to Act?

Nevertheless, being confronted with one’s own involvement in causing the suffering of sweatshop workers motivates people to help and compensate (Boster et al., 2016; Gausel et al., 2012; Iyer & Leach, 2010). Thus, perceived causal involvement could also activate the moral obligation to act, particularly when it goes against seeing oneself as a fair person (Czopp et al., 2006; Higgins, 1987), and when behavioral choices in a given situation are perceived to be relevant for one’s internalized values (Schwartz & Howard, 1981). Causal responsibility for creating another’s need also establishes a sense of connection or relatedness with the victim (Schwartz, 1977). In the case of global supply chains, where harm is carried out indirectly within a complex system, confrontations may raise awareness about the connection between consumers and sweatshop workers. By creating a sense of relatedness to those in need, causal responsibility promotes the perceived responsibility to relieve that need (Schwartz & Ben David, 1976). Such a sense of responsibility to become involved is a precondition for the activation of moral obligation (Schwartz, 1977; Steg & de Groot, 2010).

## A Self-Other Asymmetry in the Pathway from Causation to Obligation?

When it comes to accepting one’s *personal* moral obligations, however, additional factors are involved that could inhibit (or strengthen) the activation of moral obligation to act. On the one hand, people might discount their own causal responsibility for sweatshop conditions while holding others accountable. We assume that causal involvement in creating a need is most likely to increase the moral obligation to remedy this need when the causal involvement is judged as morally wrong (Darley & Pittman, 2003). Perceived wrongness depends on the extent to which consumers’ involvement in global supply chains is appraised in terms of violated moral norms (Malle, 2021).

Due to the inherent ambiguity of individual consumers’ contribution to the complex causation of sweatshop labor, people may strategically apply justifications instead of moral principles such as justice, fairness, or reciprocity when evaluating their own (vs. others’) involvement (Lammers, 2012; Shalvi et al., 2015). Beliefs that rationalize global injustice and the use of sweatshop labor in particular are prevalent in self-related judgments (Paharia et al., 2013). Alternatively, mitigating contextual details (e.g., low availability of fair-trade alternatives) might be more salient than moral principles when thinking about one’s own involvement because people think about themselves in terms of low-level construal and about abstract others in terms of high-level construal (Eyal et al., 2008). Moreover, there are principal differences in the types of information people use when thinking about themselves and others (Pronin, 2008). Specifically, when making self-judgments, people ignore their actual (negative) behavior and instead refer to their internal states such as (positive) intentions, beliefs, and feelings (Pronin & Kugler, 2007).

On the other hand, perceived similarity between one’s own suffering and the suffering of an outgroup can increase prosocial behavior (Warner et al., 2014). If we have access to such similarity being perceived by a not causally involved customer who is aware of harm done to workers elsewhere, or their inner thoughts and feelings that contain empathy or guilt towards the workers, perceived moral obligation could be high irrespective of causal involvement. A precondition for this is awareness of those inner thoughts and feelings, or low *experiential distance*. Experiential distance is a dimension of construal level (Fiedler, 2007) signifying how much experience with, that is, first-hand information from, a target has been gained. While we are experientially closest to ourselves, we may usually perceive others in an experientially distant manner. This may determine differential appraisal of inner states for self and other when considering their moral obligation to act against worker exploitation. Thus, apart from a self-other asymmetry in the effect of causal involvement on moral obligation, the greater weight of internal states over information about actual behavior could result in higher judgments of moral obligation for oneself than for others, irrespective of causal involvement.

## The Present Research

The aim of this research is threefold: Firstly, we aimed to establish the effect of causal involvement on moral obligation in the area of international exploitation. Secondly, we investigate this effect with regard to self versus others as targets of judgment. Thirdly, we consider the role of experiential distance in this setting. Seven experiments were conducted to explore to what extent consumers’ causal involvement in the perpetuation of sweatshop conditions increases their moral obligation to support workers who suffer from these conditions. Below, we report the first five studies only in an aggregated meta-analysis (details are available in the online supplements). These studies deviated from their pre-registration and should thus be regarded as exploratory (Wagenmakers et al., 2012). The two final pre-registered studies are reported in detail.

In all studies, causal involvement of the target of judgment (i.e., the self or another person, depending on study or experimental condition) was manipulated within hypothetical scenarios or alleged magazine articles. This experimental manipulation contrasted targets who were purchasing sweatshop-made products and thereby supporting companies in creating and maintaining poor working conditions with uninvolved bystanders. The potential helping behaviors to which the moral obligation to act referred included relatively unspecific actions in support of the factory workers as well as more specific acts of donating to improve the lives of the workers.

All studies were approved by the ethics committee of the Department of Psychology, University of Mainz (reference number: 2021-JGU-psychEK-S027).

## Internal Reanalysis of Exploratory Studies S1–S5

To measure the basic effect of causal involvement on moral obligation and the role of Target (Self vs. Other) in this effect, we initially conducted five studies. In all of these studies, vignette manipulations conveyed a story about a target individual consumer or consumer group who were either causally responsible or not causally responsible for bad working conditions of the manufacturers of the consumed product. Participants were then asked to indicate the causal involvement of the target and the moral obligation perceived for either themselves or the target(s). Due to inconclusive results, we conducted an informative mini meta-analysis across the individual conditions of these studies. The full text study reports for each of these experiments including details on the original aims, procedures, results and their discussion are available in the supplemental material on OSF.^1^ Table 1 provides a broad summary of the five experiments.

**Table 1:** Summary of Studies S1–S5.


STUDY	TARGET	CAUSAL INVOLVEMENT [NO INVOLVEMENT]	VIGNETTE

S1	Self vs. Other	Self: You [other tourists] vs. Other: a tourist [other tourists]	buy jewelry

S2	Self vs. Other	Self: You [other tourists] vs. Other: a tourist [other tourists]	buy jewelry

S3	Self	You [other tourists]	buy jewelry

S4	Other	Mizarcs [Dwalkhs]	buy stone furniture

S5	Self	American ingroup vs. [Chinese outgroup]	buy clothing vs. buy wooden furniture


*Note*: All studies featured a causal involvement between subjects manipulation orthogonal to the Target manipulation (if more than one Target condition).

All Studies S1–S5 made use of a vignette approach. The main purpose of Studies S1 and S2 was to explore to what extent the effect of causal involvement in harming sweatshop workers on moral obligation is smaller in self-judgments than in other-judgments. Participants were randomly assigned to one of four conditions in a 2 (role of agent: causally involved vs. *not* causally involved in creating poor working conditions) × 2 (agent: self vs. other) between subjects design. Therefore, both studies included moral obligation judgments about another person who either supported poor working conditions through their purchasing behavior or not. Study S2 was a close replication of Study S1 involving a change in the assessment of moral obligation. Specifically, moral obligation in Study S2 was conceptualized as the obligation to perform a specific and costly behavior in order to support the workers who suffer from poor working conditions: paying into a fund to raise their wage to a living wage. Study S3 used the same vignettes as Studies S1 and S2.

Study S3 focused on judgments targeted at the self. Moreover, causal involvement of the self was conflated with agent (Self vs. Other). Participants were asked to imagine either having bought a sweatshop-made product themselves (causal involvement of the self) or having witnessed others buying such products (*no* causal involvement of the self) before rating their own moral obligation to support the suffering workers. In addition to the measures of Studies S1 and S2, potential defensive reactions (i.e., justifications of poor working conditions, a rating of the (im)morality of the act of buying) were explored. Study S4 was carried out to test whether the effect of causal involvement on moral obligation within a complex economic system including many other contributors generalized to other (i.e., third, different from the self) actors. To circumvent self-identification with the actor as thoroughly as possible, a more abstract scenario was created that located the problem of poor working conditions to a fictitious planet involving fictitious societies. Study S5 aimed to replicate Study S3—the effect of causal involvement on self-assigned moral obligation—on the societal level, as in Study S4. The context was the real-world problem of poor working conditions in the global fashion industry. Targets of judgment were again the participants themselves. It was expected that being causally involved in maintaining (vs. merely observing) poor working conditions would lead to stronger moral obligation to help the workers.

To get a precise estimate of the effect of causal involvement on perceived moral obligation, a small-scale meta-analysis including Studies S1–S5 was conducted. For all studies, the simple comparisons between the experimental conditions causal involvement and *no* causal involvement were included in the meta-analysis. In the studies that included both self- and other-judgments, the subgroups were independent and thus treated as separate studies in the meta-analysis (Borenstein et al., 2011). The meta-analysis was conducted in R (version 3.5.2; R Core Team, 2018) using the metafor package (version 2.0–0; Viechtbauer, 2010).

**Figure 1:**
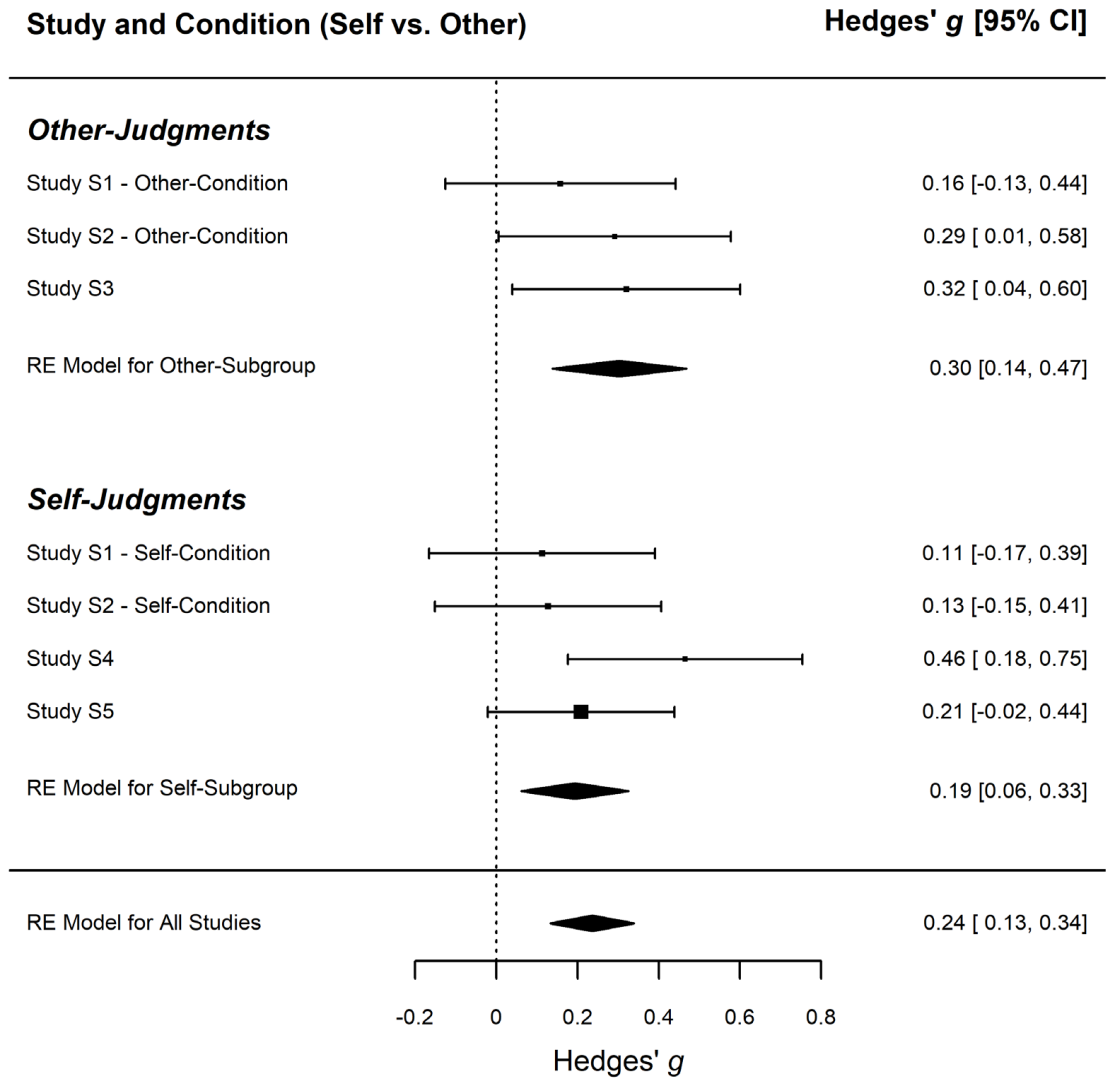
Forest Plot of the Effect Size Estimates in the Meta-analysis of Studies S3, 2, 3, S1, S2. *Note*: The forest plot is based on a random-effects model meta-analysis of the effect of causal involvement in creating and maintaining poor working conditions on the perceived moral obligation to support the suffering workers. It shows the summary effect for all comparisons included in Studies S3 to 3, S1, and S2 and the effects for the subgroups comprising other-judgments and self-judgments.

A random-effects model yielded support for a small effect of causal involvement on perceived moral obligation across all comparisons included in the investigated studies, Hedges’ *g* = 0.24, 95% CI [0.13, 0.34], *SE* = 0.05, *z* = 4.49, *p* < .001 (Figure 1), with no indication of heterogeneity of the underlying population effect, *T^2^* = 0.000, 95% CI [0.000, 0.057], *I^2^* = 0.00%, 95% CI [0.00, 74.52]. As indicated by the large confidence intervals, precision of the estimates of heterogeneity was very low.

Despite the lack of heterogeneity, we obtained separate effect size estimates of separate RE Models by target (Self vs. Other) in a mixed-effects model for descriptive reasons. The mean effect size for the RE Model for the Self-Subgroup was Hedges’ *g* = 0.19, 95% CI [0.06, 0.33], *SE* = 0.07, *z* = 2.88, *p* = .004; for the RE Model for the Other-Subgroup, it was Hedges’ *g* = 0.30, 95% CI [0.14, 0.47], *SE* = 0.08, *z* = 3.60, *p* < .001. There was no significant difference between the mean effect sizes of these subgroups, estimate = 0.11, 95% CI [–0.12, 0.34], *SE* = 0.09, *z* = 1.20, *p* = .23 (with Knapp and Hartung adjustment).

Although the hypothesis of a differential effect of causal involvement for self versus other targets received no meta-analytic support, we speculated whether participants might have easily identified with the other person (an individual tourist) in the condition involving other-judgments in Studies S1 and S2. When other-judgments were about abstract individuals from a fictitious society (Study S4), the effect size was higher than in self-judgments regarding the analogous real-world problem of poor working conditions in the garment industry (Study S5). The following study tested this difference in a purely confirmatory manner.

## Study 1

The purpose of Study 1 was to perform a confirmatory test whether the effect of causal involvement on moral obligation depends on whether one judges oneself or someone else. Based on the reanalysis of our previous findings, we pre-registered two hypotheses:^2^ A) Being involved in causing (vs. merely observing) poor working conditions leads to stronger moral obligation to support the suffering workers. B) The effect of causal involvement is smaller when considering one’s own behavior compared to the judgment about others.

### Method

#### Participants

This study included 778 American participants (379 female, 393 male, 3 other, 3 did not indicate). Participants were recruited from MTurk. They were between 18 and 75 years old (*M* = 37.73, *SD* = 11.78). Thirty-two other participants who met the exclusion criteria were excluded from the sample. The sample size of *N* = 778 provided 90% power to detect a small effect of Cohen’s *f* = 0.12.

#### Design, Materials, and Procedure

Participants were randomly assigned to one of four conditions in a 2 (Involvement: causal [ingroup caused poor working conditions] versus *not* causal [outgroup caused poor working conditions]) × 2 (Target of judgment: self versus other [i.e., an average Chinese person]) between subject design.

The session started with a reading task that asked participants to read a short text, ostensibly taken from a recent magazine. Depending on experimental condition, participants either read that Chinese or American consumption has harmful consequences for workers in Indonesia. Participants in the *causal involvement* condition read that many Americans (i.e., participants’ ingroup) are buying pieces of clothing that are produced under poor working conditions, even though most of them know about the problem. By contrast, participants in the *no causal involvement* condition read about the purchase of wooden furniture by Chinese consumers involving the same problems. The text introduced both national groups but emphasized that only the consumers mentioned in the text (Chinese vs. Americans) but not the other group contributed to poor working conditions in Indonesia.

It was decided to also vary the product (clothes vs. furniture) between conditions to make it less likely for participants in the *no causal involvement* condition to see parallels to their own behavior. The text argued that American [Chinese] consumers were ‘supporting companies in creating and maintaining these poor working conditions.’ The last paragraph of the text informed participants that they could effectively help the workers by donating to ‘effective organizations that are doing good work to support the rights of the factory workers in Indonesia and other South Asian countries.’

After the reading task, participants completed the dependent measures on either ‘an average Chinese person who is aware about the poor working conditions’ or the participant themselves, depending on experimental condition. The key dependent variable was moral obligation (Cronbach’s α = .84), which was assessed by a three-item measure on a 7-point scale (e.g., ‘To what extent do you think you [the Chinese] are morally obligated to act to improve the situation of the workers?’; full wording on OSF). In addition, perceived causal responsibility was assessed with one item (‘To what extent do you think you [the Chinese] contributed to supporting the poor working conditions?’; 1 = *not at all contributed* to 7 = *very much contributed*). Finally, participants completed a manipulation check and indicated demographic information before being thanked and debriefed.

### Results

As expected, perceived causal responsibility was higher when the target of judgment was a member of the group that was involved in causing the poor working conditions, with a large effect, *F*(1, 774) = 125.30, *p* < .001, η_p_^2^ = .139, 90% CI [0.104, 0.177]. Although there was evidence for a small interaction effect, *F*(1, 774) = 8.64, *p* = .003, η_p_^2^ = 0.011, 90% CI [0.002, 0.026], the main effect of causal involvement on perceived causal responsibility was of medium size in the case of self- (Hedges’ *g_s_* = 0.57, 95% CI [0.39, 0.77]) and large in other-judgments (Hedges’ *g_s_* = 1.05, 95% CI [0.84, 1.26]).

**Figure 2:**
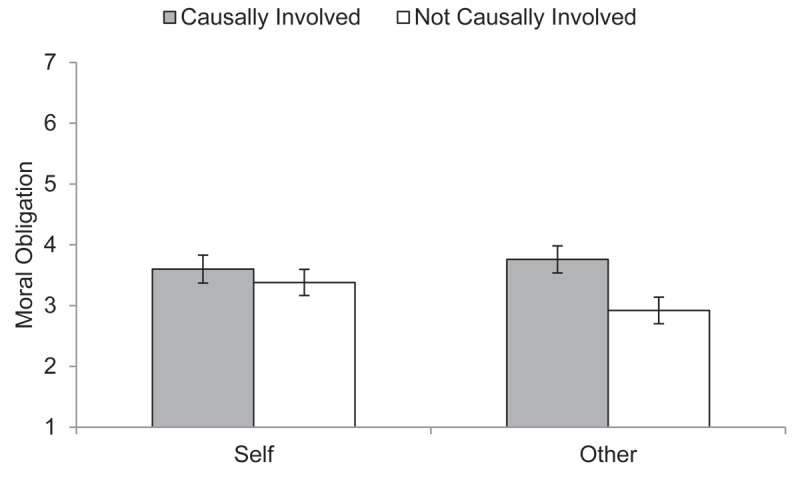
Moral Obligation Judgments in Study 1. *Note*: Mean moral obligation judgments (with 95% CIs) as a function of experimental group (role of target: causally involved vs. *not* causally involved in creating poor working conditions × 2 target: self vs. other).

Moral obligation scores showed the predicted (small-to-medium) main effect of causal involvement, *F*(1, 774) = 21.69, *p* < .001, η_p_^2^ = 0.027, 90%^3^ CI [0.011, 0.049] (Figure 2; see Tables A1, A4, A5 in the Appendix for descriptives and ANOVAs) as well as the predicted interaction with a small effect, *F*(1, 774) = 7.56, *p* = .006, η_p_^2^ = 0.010, 90% CI [0.002, 0.024]. When judging others, moral obligation ratings were higher for the causally involved than for the *not* causally involved targets, medium effect of Hedges’ *g_s_* = 0.53, 95% CI [0.33, 0.74]. When judging the self, moral obligation ratings were not substantially higher in the case of causal involvement, small effect of Hedges’ *g_s_* = 0.14, 95% CI [–0.06, 0.33].

### Discussion

Study 1 provided evidence that causal involvement in harming sweatshop workers has divergent effects on moral obligation dependent on whether judgments are about oneself or another person. Others were judged to be more morally obligated to support the workers when they have contributed to supporting these conditions compared to being a neutral bystander. In contrast, the self was judged to be equally obligated irrespective of being involved in causing the poor situation to some extent or not. The results support the idea that, in general, a person’s involvement in causing harm matters for how morally obligated to help they are judged, even in the case of indirect harm within global supply chains. Yet, when judgments are about the self, being causally involved or not ceases to matter.

One aspect of this pattern might deserve future scrutiny, though. Although our original reasoning (and the relevant literature) was built on the notion that moral obligation for the self is *lower* than for others in the case of causal responsibility, the observed interactive pattern seems to result from the fact that perceived moral obligation for others is lower in cases of no causal responsibility. Others are left ‘off the hook’ more easily than the self. Put differently, perceived *own* moral obligation is high independent of causal responsibility. If one’s own perception of moral obligation does not depend on causal responsibility to the same extent as for others, it may be that moral obligation is borne out of one’s feelings of empathy with the workers or guilt for being in comparably privileged position. Study 2 tested this possibility directly.

## Study 2

Study 2 was conducted as a preregistered replication of the main effect of causal involvement on judgments of moral obligations. In addition, we sought to test the mediation of this effect by *experiential distance*, that is, access to inner thoughts and feelings, a variable we suspect to be responsible for the previously observed self-other differences and the finding that in the absence of causal involvement, others were left ‘off the hook’ of moral obligation more easily than the self.

Study 1 indicated that one’s own moral obligation is perceived to be high irrespective of causal involvement, while no causal involvement leads to lower perceived moral obligation of others. In line with these results, access to our own internal thoughts and feelings could provide access to feelings of guilt towards poorly treated workers, for example, for our own privilege and their (not self-inflicted) misfortune, even when we are not causally responsible for their misery. Thus, we may still feel morally obligated to act. For ‘others,’ we usually do not have the same access to these internal states, so we may consider others less morally obligated when they are not involved causally. Thus, we manipulated *experiential distance* to an ‘other’ average US-American customer as target between conditions. This way, we also circumvented possible confounds in comparing ingroup versus outgroup targets in previous self-other target manipulations.

Furthermore, we measured perceived moral obligation by means of the established scale by Sabucedo et al. (2018) and explored whether causal involvement and perceived moral obligation affected donation behavior. We pre-registered two main hypotheses:^4^ A) Being involved in causing (vs. merely observing) poor working conditions leads to stronger moral obligation to support the suffering workers. B) The effect of causal involvement is smaller when considering an experientially close other’s behavior compared to the judgment about an experientially distant other.

### Method

#### Participants

*N* = 811 American participants completed the study on MTurk. Preregistered criteria led to the exclusion of *n* = 283 datasets (detailed breakdown see online supplements on OSF). The final sample of *N* = 528 (309 female, 214 male, 4 diverse, 1 did not indicate) were between 18 and 77 years old (*M* = 41.56, *SD* = 13.01). The target sample size of *N* = 800 was determined in advance to find the interaction of causal involvement × self-other (Study 1: η_p_^2^ = .027) and the meta-analytical main effect of causal involvement on moral obligation (Hedges’ *g* = .24) with 90% power. The final dataset still had 75% power to detect the preregistered interaction and 78% power to detect the meta-analytical main effect.

#### Design, Materials, and Procedure

Participants were randomly assigned to one of four conditions in a 2 (Involvement: ingroup caused poor working conditions vs. outgroup caused poor working conditions) × 2 (Target of judgment: close other vs. distant other) between subjects design. The study started with a reading task (similar to Study S5; see supplemental material). Depending on experimental condition, participants read that the global (implying also the US-American ingroup) consumption of cocoa products or exclusively Asian (mainly the Chinese outgroup) consumption of azuki bean products has harmful consequences for farmers of the respective raw beans in West Africa. The text on cocoa was a written summary of a docuseries episode (‘Rotten’) to provide a realistic manipulation of causal involvement. It was adapted for the *not causally involved* condition by replacing ‘cocoa’ with ‘azuki bean.’ A few lines were added to emphasize that azuki beans were almost exclusively traded and consumed by Asians, especially the Chinese, to make the point that US-Americans were not causally involved. After the reading task, participants completed a short text comprehension manipulation check. The questions differed between conditions only in the kind of bean referred to.

In the second, cross-cutting manipulation of experiential distance, participants were instructed to ‘try to put yourself in the position of the described protagonist. What are the protagonist’s interests, intentions, and feelings?’ (experientially close condition) or ‘try to create a visual mental image of the scene described in the text’ (experientially distant condition). The described scene either was an average US-American customer shopping for chocolate (and choosing a non-fair-trade bar) in the causal involvement condition or canned tomatoes in the no causal involvement condition. After reading the shopping scene, they were asked to note their impressions in an open response. In both conditions, a statement of customer awareness about poor working conditions was included right before the moral obligation measure.

After another manipulation check, participants completed measures of perceived experiential distance (11 items, e.g. ‘I can imagine why the customer made this decision,’ Cronbach’s α = .88) and psychological distance (1 item, adapted from Pronin & Ross, 2006, ‘What was your visual perspective on the scene?’ [1-mostly the customer’s point of view, 7-mostly my point of view), and then the two main dependent measures of causal involvement (4 items, adapted from Messer & Imhoff, 2021, e.g., ‘The customer is causally responsible for what is happening to [cocoa farmers/ azuki bean farmers] in the global South. [1-strongly disagree, 7-strongly agree],’ Cronbach’s α = .94) and moral obligation (5 items, adapted from Sabucedo et al., 2018, e.g. ‘To mobilize against the poor working conditions of West Africa [cocoa/ azuki bean] farmers constitutes a moral obligation to the US-American customer. [1-totally disagree, 5-totally agree],’ Cronbach’s α = .88). Afterwards, participants could choose what amount of their bonus payment they wanted to donate to a foundation concerned with West African cacao/Azuki worker’s rights. After completing demographic questions and questions about their cacao/Azuki bean consumption, their previous knowledge about the issue and data quality, they were debriefed (and in the Azuki condition informed that their donation would go towards cocoa workers instead).

### Results

The dependent measures were subjected to separate 2 (target of judgment: causally involved vs. *not* causally involved in creating poor working conditions) × 2 (target of judgment: close other vs. distant other) ANOVAs. As expected, perceived causal responsibility was considerably higher when the target of judgment was a member of the group that was involved in causing the poor working conditions, *F*(1, 524) = 81.70, *p* < .001, η_p_^2^ = .135, 90% CI [0.093, 0.180]. The interaction effect did not become significant, although the effect size was similarly small as in Study 1, *F*(1, 524) = 3.42, *p* = .065, η_p_^2^ = 0.006, 90% CI [0.000, 0.023]. Participants acknowledged causal responsibility both in the judgment of close (medium effect of Hedges’ *g_s_* = 0.62, 95% CI [0.39, 0.85]) and distant others (large effect of Hedges’ *g_s_* = 0.98, 95% CI [0.71, 1.26]).

**Figure 3:**
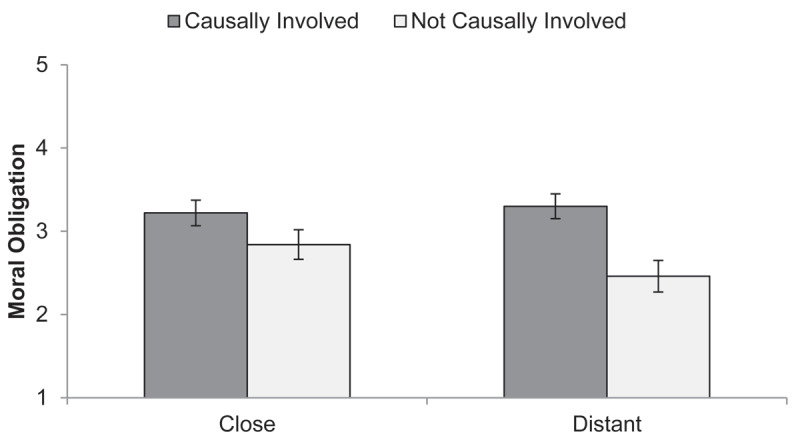
Moral Obligation Judgments in Study 2. *Note*: Mean moral obligation judgments (with 95% CIs) as a function of experimental group (role of target: causally involved vs. *not* causally involved in creating poor working conditions × 2 target: close vs. distant other).

Moral obligation scores showed the predicted main effect of causal involvement, which was medium-to-large, *F*(1, 524) = 49.74, *p* < .001, η_p_^2^ = 0.087, 90% CI [0.052, 0.127] (Figure 3; see Tables A2, A6, A7 in the Appendix for descriptives and ANOVAs). In addition, the 2 (Involvement: causal vs. not causal) × 2 (Target: close other vs. distant other) interaction, *F*(1, 524) = 6.82, *p* = .009, η_p_^2^ = 0.013, 90% CI [0.002, 0.033] showed that the effect of causal involvement on perceived moral obligation was lower when the customer was perceived as experientially close. Moral obligation ratings were higher for the causally involved than for the *not* causally involved targets both when judging distant others, large Hedges’ *g*_s_ = 0.91, 95% CI [0.64, 1.18], and when judging close others, small-to-medium Hedges’ *g_s_* = 0.37, 95% CI [0.14, 0.60], but judging from the nonoverlapping confidence intervals, the effect was larger for the judgment of distant others.

The two following mediation models featuring perceived causal involvement and perceived experiential distance as mediators provide additional manipulation checks for the findings above. As predicted, the results were compatible with a mediation of the effect of causal involvement on moral obligation via perceived causal involvement. The standardized regression coefficient between causal involvement and perceived causal involvement was statistically significant (*β* = .73, *t*(526) = 8.90, *p* < .001, *R*^2^ = .13, *F*(1, 526) = 79.19, *p* < .001), as was the standardized regression coefficient between perceived causal involvement and moral obligation (*β* = .65, *t*(526) = 18.83, *p* < .001, *R*^2^ = .45, *F*(2, 525) = 215.83, *p* < .001). The standardized indirect effect was *β* = .47. The unstandardized indirect effect based on 10,000 bootstrapped samples was significant (b = .49, 95% CI [.37, .61]). The effect of causal involvement on moral obligation was reduced from *β* = .57, *p* < .001, to *β* = .10, *p* = .17 when perceived causal involvement was entered as an additional predictor.

The effect of the experiential distance manipulation on moral obligation was also compatible with a mediation via perceived experiential distance, but not psychological distance. The standardized regression coefficients between experiential distance and both perceived experiential distance (*β* = .46, *t*(246) = 3.73, *p* < .001, *R*^2^ = .05, *F*(1, 246) = 13.91, *p* < .001) and psychological distance (*β* = 1.06, *t*(246) = 9.81, *p* < .001, *R*^2^ = .28, *F*(1, 246) = 96.19, *p* < .001) were statistically significant, as was the standardized regression coefficient between perceived experiential distance and moral obligation (*β* =. 16, *t*(244) = 2.45, *p* = .01, *R*2 =.05, *F*(3, 244) = 4.72, *p* = .003), but not psychological distance and moral obligation (*p* = .76). The standardized indirect effect of experiential distance was *β* = .07. The unstandardized indirect effect based on 10,000 bootstrapped samples was significant (b = .08, 95% CI [.01, .17]). The effect of experiential distance on moral obligation in the absence of causal involvement was reduced from *β* = .38, *p* = .005, to *β* = .30, *p* = .03, when perceived experiential distance was entered as an additional predictor.

While donation behavior was correlated with both perceived causal involvement (*r* = .13, *p* = .004) and moral obligation (*r* = .19, *p* < .001), participants did not donate more when their causal involvement was implied by framing their ingroup of US-Americans as causally involved (*t*(525) = 1.38, *p* = .17). The 2 (Involvement: causal vs. not causal) × 2 (Target: close other vs. distant other) interaction on donation behavior was also not significant, *F*(1, 523) = 0.09, *p* = .76, η_p_^2^ < 0.001. However, there was a significant indirect effect of causal involvement through moral obligation (*β* = .57, *t*(525) = 6.79, *p* < .001, *R*^2^ = .08, *F*(1, 525) = 46.06, *p* < .001) on donation behavior (*β* = .18, *t*(525) = 4.12, *p* < .001, *R*^2^ =.03, *F*(2, 525) = 9.46, *p* < .001). As this indirect effect was not qualified by a total effect, the b path of the model might have become significant not due to the hypothesized indirect effect, but due to a common confound of perceived moral obligation and donation behavior. By means of a LOVE (left out variable error) analysis (Mauro, 1990), the size of such a confound necessary to explain the b path can be estimated. An unobserved confounding variable that correlates approximately *r* = .57 with both perceived moral obligation and donation behavior would be necessary to reduce the observed indirect effect to zero. As such a strong confound is unlikely to exist, the hypothesized indirect effect gains further credibility (detailed analysis in online supplement on OSF).

## Discussion

Study 2 confirmed that the influence of causal involvement on moral obligation is dependent on the target of judgment. In Study 1, in the causal involvement condition, relative to the no causal involvement condition, higher moral obligation was assigned to others but not to the self. Study 2 provided evidence for a possible explanation for this self-other discrepancy. While again, others were judged to be more morally obligated to the workers when causal involvement was present, this effect was attenuated when participants felt experientially close to the target of judgment. Thus, (perceived) access to the inner thoughts and feelings of the judged consumer reduced the impact of causal involvement on their perceived moral obligation. This may explain the previously discovered self-other discrepancy. However, there was still a small main effect of causal involvement on moral obligation in the close other condition. While it could be argued that even the experientially closest other will never be as close to us as we are close to ourselves, there may also be other processes at play which we discuss in the general discussion.

Two mediation analyses underpinned the construct validity of both the causal involvement manipulation and the experiential distance manipulation. Furthermore, there was a small but significant indirect effect of causal involvement through moral obligation to act on actual donation behavior. Thus, at least for others, it may be more acceptable to not act against poor worker conditions on the other end of the supply chain when they are not causally involved, because they seem less morally obligated from afar.

The effect size of the predicted interaction effect on moral obligation was of similar magnitude as the one found in Study 1, Cohen’s *f* = 0.11 (Study 1: Cohen’s *f* = 0.10). The results solidify the idea that even an indirect causal involvement through participation in a global supply chain increases perceived moral obligation to help the workers. However, the results also point to and clarify a boundary condition of this effect: Internal thoughts and feelings may override the propensity to perceive less moral obligation if causal involvement is not present.

## General Discussion

We investigated how individual participation in global supply chains involving poor working conditions affects judgments of moral obligation. The present research shows that people consider individual causal involvement to be relevant, even when harm is carried out indirectly through a chain of numerous actors. In general, participants saw individuals who contributed to causing sweatshop conditions as more morally obligated to act than uninvolved others who merely witnessed the suffering of the workers.

However, the hope of increasing consumers’ own sense of moral obligation to act by confronting them with their own involvement in causing sweatshop conditions received no support. Although causal involvement moderately increased ratings of moral obligation when participants made judgments about abstract others (Studies S4,1 and 2), the effect of causal involvement was small to non-existent when facing their own complicity in maintaining sweatshop conditions (Studies S1, S2, S3, S5, and 1). Interestingly, people acknowledged their own causal responsibility almost as much as they accepted the causal responsibility of others. In their moral obligation judgments, however, they neglected information about causal responsibility in the case of self-judgments.

There are several possible reasons why this was the case. First, confrontation with the own causal involvement might have failed to establish a sense of relatedness with the sweatshop workers, which is a necessary step in the activation of moral obligation (Schwartz, 1977). We consider this explanation unlikely because the confrontations led to a strong increase in acceptance of causal responsibility. Second, an existing effect of causal involvement could have been masked by a parallel process, such as an increase in moral outrage in the *no* causal involvement condition where third parties (other tourists or Chinese consumers) were made responsible for the workers’ plight. However, additional analyses in Study S5 revealed that the information that others are involved in harming sweatshop workers did not increase moral outrage (see online supplemental materials on OSF). Third, participants might have justified their own participation in an economic system based on sweatshop conditions. Yet, although people are more likely to endorse justifications when considerations about sweatshop conditions are self-relevant (Paharia et al., 2013), the present results showed no or only a small increase in exculpatory tendencies in response to confrontation with own causal involvement (Studies S1, S2, S3).

Rather, we suggest that the observed self-other asymmetry might be explained by the general tendency of people to value thoughts and ignore information about actual behavior when making *self*-judgments but to rely on actual behavior in *other*-judgments (Pronin & Kugler, 2007). In other-judgments, supporting sweatshop workers is seen as a generous but not obligatory act if the behavioral information indicates that the target person is *uninvolved*, whereas it is regarded as a moral obligation, if the behavioral information indicates that others are *involved* in causing harm. Possibly, people hold causally involved others responsible for their involvement because they perceive their behavior as negligent, or even construe their intentions as malevolent (Klein & Epley, 2017). In contrast, people consider their *own* involvement irrelevant when judging their moral obligation. Irrespective of this behavioral information, they base their self-judgments on their internal states such as feelings, beliefs, and intentions (Pronin & Kugler, 2007). Even in the case of causal responsibility for harm, people may see themselves as being guided by ethical intentions (Klein & Epley, 2017). Study 2 provides evidence that confirms this account in the context of ascription of moral obligation.

Unexpectedly, we observed some indications that, although people seem to be more lenient in what their own (vs. others’) causal involvement implies for the moral obligation to act, they generally seem to have higher expectations regarding their own moral obligation compared to that of others. Moral obligation tended to be comparably high in the case of self-judgments and decreased in the case of no causal involvement only in other-judgments (in fact, there was a main effect of self versus other in Studies S1 and S2, and a tendency in Study 1). Such a self-other main effect in moral obligation judgments may be present in addition to the asymmetry in the effect of causal responsibility discussed above. This could have resulted in the observed overall pattern, specifically also the small main effect of involvement on moral obligation in close others in Study 2. Possibly, such higher expectations of the self reflect the anticipated negative self-evaluations in case of not helping. This interpretation fits well with research showing that people *believe* that they would feel worse than others after acting immorally (Klein & Epley, 2017).

This sort of ceiling effect could not only affect perceptions of own moral obligation, but also perceptions of own causal involvement. Participants may strongly believe in general that *they* (in contrast to others) are causally responsible for harming sweatshop workers, irrespective of any experimental manipulation of causal involvement. This may leave no room for an experimental effect of causal responsibility to further increase the perception of participants’ own causal involvement. Consequently, such a ceiling effect would also inhibit an experimentally induced effect of causal involvement on moral obligation in the Self condition selectively—which is the pattern we find in in Study 1.

This pattern of results can also be interpreted through the lens of the four Models of Helping and Coping (Brickman et al., 1982). It states that whether or not a person is assigned (a) the responsibility for a problem and (b) the responsibility for a solution, different views on the person in need emerge. The Moral Model states that the person who caused a problem is also responsible to solve it. In the context of the present studies, this would imply that causal involvement leads to moral obligation. The Compensatory Model states that a person can feel responsible to solve a problem, even though they did not cause it. Our pattern of results may indicate that the Moral Model is applied equally to self, close others and distant others. In contrast, people may apply the Compensatory Model more to themselves than to close others, and more to close others than to distant others. The Compensatory Model holds the highest potential for social rewards: Despite not having caused the problem, a person feels responsible for helping to resolve it. Earning this kind of respect could be a motivation that is also more accessible if a person has access to the target’s inner thoughts and feelings.

Lastly, our results are also in line with the Theory of Dyadic Morality (TDM; Schein & Gray, 2018). According to the TDM, perceived own moral obligation does not depend on own causal involvement—perceiving any clear causal perpetrator is enough. Conversely, the perceived causal involvement of another person may strengthen the perceived moral obligation of this other person because it indicates individual agency and control (Schein & Gray, 2018).

The present research makes a descriptive and explanatory contribution to the normative debate about whether individual citizens in the global North should feel responsible for global poverty and what the benefits of confrontations could be (e.g., Lichtenberg, 2010; Pierik, 2013; Pogge, 2014). Our findings about how people actually perceive the implications of their causal involvement show that in general, people do infer greater moral obligation from causal involvement in maintaining global injustice, but less so when it comes to their *own* participation. Future research may show whether the *general* acceptance of the moral implications of causal responsibility can be used to increase this effect in individuals’ *own* feelings of moral obligation.

Ending poverty and promoting decent work for all have been targeted by the 193 member states of the United Nations General Assembly in their declaration of the Sustainable Development Goals (SDGs) in 2015. Reaching these goals critically depends on the development of sustainable consumption patterns by citizens in the global North. Although politicized identities, emotions, and efficacy beliefs can help explain why people act against global poverty (e.g., Iyer & Leach, 2010; Thomas & McGarty, 2018), less is known about the consequences of consumers facing their complicity in maintaining sweatshop conditions. Our research suggests that the effect of causal involvement on perceived moral obligation cannot be applied unconditionally on scenarios of international exploitation, let alone be used in social interventions, as other processes like reliance on internal states may run counter to the suggested effect. On the other hand, perceived moral obligation could be supported by promoting self-reflection in consumers or decreasing their experiential distance towards causally involved individuals or groups if the consumers themselves are merely bystanders. While our results could only suggest some mechanisms that *do not* work, they may still be valuable in directing future pursuits of what *does* work to increase the moral obligation to act in consumers at the receiving end of a long and winding global supply chain.

## Data Accessibility Statement

Our preregistrations for Study 1^5^ and Study 2^6^ included the study design, planned sample size, inclusion/exclusion criteria, and planned analyses, but no analysis plan. In Studies 1 and 2, we report all preregistered analyses in the main body of the manuscript. There were no deviations from the preregistered analysis plan. All materials, de-identified raw data, and analysis scripts including additional analyses can be found on the Open Science Framework under https://osf.io/uxpvg/?view_only=5782c590514541c595e0be19951ce322. For all studies, exclusion criteria and sample sizes were set before data collection began. Sample sizes were planned to have at least 100 (Studies 1 and 2: 200) participants per cell. Sensitivity analyses are reported in the method sections. Where not indicated differently, data analysis was conducted in SPSS, version 23 (IBM Corp., 2015).

## Footnotes

https://osf.io/cdapf?view_only=5782c590514541c595e0be19951ce322.

https://aspredicted.org/blind.php?x=hq56ey.

Note that 90% CIs are reported in line with current recommendations (Lakens, 2014). Contrary to effect size estimates like Cohen’s d or Hedges’ g, squared estimates like r^2^ or η_p_^2^ cannot take on negative values. Therefore, 95% CIs could include zero even in the presence of a statistically significant result and start at 0 even when the result is n.s. (as the point estimates cannot be negative, their lower bounds cannot, either). Thus, CIs for squared effect sizes of n.s. results should be reported as [.00; upper bound].

https://aspredicted.org/blind.php?x=38hv8r.

https://aspredicted.org/blind.php?x=hq56ey.

https://aspredicted.org/blind.php?x=38hv8r.

## Appendix

### Means and Standard Deviations for All Analyses in All Studies

**Table A1:** Means and Standard Deviations (in Parentheses) of all Dependent Variables as a Function of Causal Involvement of the Target of Judgment and Self- versus Other-Judgment in Study 1.

DEPENDENT VARIABLE	SELF		OTHER	
	
	CAUSALLY INVOLVED	NOT CAUSALLY INVOLVED	CAUSALLY INVOLVED	NOT CAUSALLY INVOLVED

Moral Obligation	3.60 (1.63)	3.38 (1.54)	3.76 (1.60)	2.92 (1.54)

Causal Responsibility	3.67 (1.83)	2.63 (1.81)	4.67 (1.64)	2.89 (1.73)

*Note*: All dependent variables range from 1 to 7.

**Table A2:** Means and Standard Deviations (in Parentheses) of All Dependent Variables as a Function of Causal Involvement of the Target of Judgment and Self- versus Other-Judgment in Study 2.

DEPENDENT VARIABLE	CLOSE OTHER		DISTANT OTHER	
	
	CAUSALLY INVOLVED	NOT CAUSALLY INVOLVED	CAUSALLY INVOLVED	NOT CAUSALLY INVOLVED

Moral Obligation	3.22 (1.00)	2.84 (1.06)	3.30 (0.82)	2.46 (1.03)

Causal Responsibility	4.04 (1.61)	2.99 (1.79)	4.13 (1.47)	2.53 (1.77)

*Note*: Causal Responsibility ranges from 1 to 7 on a 7-point scale, Moral Obligation ranges from 1 to 5 on a 5-point scale.

**Table A3:** Summary of Studies in This Line of Research.

STUDY	TARGET	CAUSAL INVOLVEMENT [NO INVOLVEMENT]	VIGNETTE

S1	Self vs. Other	Self: You [other tourists] vs. Other: a tourist [other tourists]	buy jewelry

S2	Self vs. Other	Self: You [other tourists] vs. Other: a tourist [other tourists]	buy jewelry

S3	Self	You [other tourists]	buy jewelry

S4	Other	Mizarcs [Dwalkhs]	buy stone furniture

S5	Self	American ingroup vs. [Chinese outgroup]	buy clothing vs. buy wooden furniture

1	Self vs. Other	American ingroup vs. [Chinese outgroup]	buy clothing vs. buy wooden furniture

2	Close vs. Distant Other	Cocoa [Azuki beans]	buy cocoa products vs. Azuki bean products

*Note*: All studies featured a causal involvement between subjects manipulation orthogonal to the Target manipulation (if more than one Target condition).

**Table A4:** Fixed-Effects ANOVA Results of Study 1 Using Causal Responsibility as a Criterion.

PREDICTOR	SUM OF SQUARES	*df*	MEAN SQUARE	*F*	*p*	η_p_^2^	η_p_^2^ 90% CI [LL, UL]

(Intercept)	9351.64	1	9351.64	3036.18	<.001	.797	[.78, .81]

Involvement	385.92	1	385.92	125.29	<.001	.139	[.10, .18]

Target	78.01	1	78.01	25.33	<.001	.032	[.01, .05]

Involvement × Target	26.62	1	26.62	8.64	.003	.011	[.00, .03]

Error	2383.97	774	3.08				

*Note*: LL and UL represent the lower-limit and upper-limit of the partial η_p_^2^ confidence interval, respectively.

**Table A5:** Fixed-Effects ANOVA Results of Study 1 Using Moral Obligation as a Criterion.

PREDICTOR	SUM OF SQUARES	*df*	MEAN SQUARE	*F*	*p*	η_p_^2^	η_p_^2^ 90% CI [LL, UL]

(Intercept)	9066.11	1	9066.11	3646.37	<.001	.825	[.81, .84]

Involvement	53.92	1	53.92	21.69	<.001	.027	[.01, .05]

Target	4.29	1	4.29	1.73	.19	.002	[.00, .01]

Involvement × Target	18.79	1	18.79	7.56	.006	.010	[.00, .02]

Error	1924.43	774	2.49				

*Note*: LL and UL represent the lower-limit and upper-limit of the partial η_p_^2^ confidence interval, respectively.

**Table A6:** Fixed-Effects ANOVA Results of Study 2 Using Causal Responsibility as a Criterion.

PREDICTOR	SUM OF SQUARES	*df*	MEAN SQUARE	*F*	*p*	η_p_^2^	η_p_^2^ 90% CI [LL, UL]

(Intercept)	6046.40	1	6046.40	2187.47	<.001	.807	[.79, .82]

Involvement	225.83	1	225.83	81.70	<.001	.135	[.09, .18]

Target	4.40	1	4.40	1.59	.21	.003	[.00, .02]

Involvement × Target	9.46	1	9.46	3.42	.07	.006	[.00, .02]

Error	1448.39	524	2.76				

*Note*: LL and UL represent the lower-limit and upper-limit of the partial η_p_^2^ confidence interval, respectively.

**Table A7:** Fixed-Effects ANOVA Results of Study 2 Using Moral Obligation as a Criterion.

PREDICTOR	SUM OF SQUARES	*df*	MEAN SQUARE	*F*	*p*	η_p_^2^	η_p_^2^ 90% CI [LL, UL]

(Intercept)	4509.87	1	4509.87	4653.78	<.001	.899	[.89, .91]

Involvement	48.205	1	48.21	49.74	<.001	.087	[.05, .13]

Target	2.882	1	2.88	2.97	.09	.006	[.00, .02]

Involvement × Target	6.607	1	6.61	6.82	.01	.013	[.00, .03]

Error	507.796	524	.97				

*Note*: LL and UL represent the lower-limit and upper-limit of the partial η_p_^2^ confidence interval, respectively.

## Author Information

The initial idea for this paper was conceived by the second author and the third author, who also designed Studies S1–S5 and Study 1. Study 2 was designed by the third author and the first author. Data was collected, analyzed and interpreted by the second author and the first author for these studies, respectively. The third author also interpreted the data. The initial manuscript was drafted by the second author, it was substantially revised by the first author. All authors revised and approved the final version for submission.

Correspondence concerning this article should be addressed to the first author.
